# Evaluation of race and ethnicity disparities in outcome studies of *CYP2C19* genotype-guided antiplatelet therapy

**DOI:** 10.3389/fcvm.2022.991646

**Published:** 2022-08-23

**Authors:** Anh B. Nguyen, Larisa H. Cavallari, Joseph S. Rossi, George A. Stouffer, Craig R. Lee

**Affiliations:** ^1^Division of Pharmacotherapy and Experimental Therapeutics, Eshelman School of Pharmacy, University of North Carolina at Chapel Hill, Chapel Hill, NC, United States; ^2^Department of Pharmacotherapy and Translational Research and Center for Pharmacogenomics and Precision Medicine, University of Florida, Gainesville, FL, United States; ^3^Division of Cardiology, School of Medicine, University of North Carolina at Chapel Hill, Chapel Hill, NC, United States

**Keywords:** cytochrome P450 (CYP), precision medicine, clopidogrel, pharmacogenomics, race and ethnicity

## Abstract

Dual antiplatelet therapy with a P2Y_12_ inhibitor (clopidogrel, prasugrel, or ticagrelor) and aspirin remains the standard of care for all patients undergoing percutaneous coronary intervention (PCI). It is well-established that patients carrying *CYP2C19* no function alleles have impaired capacity to convert clopidogrel into its active metabolite and thus, are at higher risk of major adverse cardiovascular events (MACE). The metabolism and clinical effectiveness of prasugrel and ticagrelor are not affected by *CYP2C19* genotype, and accumulating evidence from multiple randomized and observational studies demonstrates that *CYP2C19* genotype-guided antiplatelet therapy following PCI improves clinical outcomes. However, most antiplatelet pharmacogenomic outcome studies to date have lacked racial and ethnic diversity. In this review, we will (1) summarize current guideline recommendations and clinical outcome evidence related to *CYP2C19* genotype-guided antiplatelet therapy, (2) evaluate the presence of potential racial and ethnic disparities in the major outcome studies supporting current genotype-guided antiplatelet therapy recommendations, and (3) identify remaining knowledge gaps and future research directions necessary to advance implementation of this precision medicine strategy for dual antiplatelet therapy in diverse, real-world clinical settings.

## Introduction

Cardiovascular disease, including coronary artery disease and stroke, remains among the leading cause of death in the United States (U.S.) and worldwide (1). In the U.S., the percentage of all deaths caused by cardiovascular disease in 2019 was approximately 32% in Black, 28% in Hispanic, and 30% in White individuals (1). Although significant advances in the diagnosis and treatment of cardiovascular disease have occurred over the past several decades, racial and ethnic disparities in cardiovascular disease prevalence and mortality continue to persist between White populations and both Black and Hispanic populations (2–4).

Racial and ethnic minority groups remain underrepresented in cardiovascular clinical trials, which has contributed to an incomplete understanding of these health disparities (5). According to the U.S. Food and Drug Administration’s Center for Drug Evaluation and Research (CDER), of the 58,998 participants who participated in FDA registered cardiovascular trials from 2015 to 2016, only about 3% identified as Black or African American and about 8.5% identified as Hispanic (6). Lack of diversity in clinical trials results in lack of adequate data to rigorously evaluate the safety and efficacy of therapeutic interventions within underrepresented racial and ethnic minority groups (5). Diverse racial and ethnic representation is crucial for demonstrating generalizability of clinical trial results to more diverse real-world clinical settings, and to ensure equity when developing therapeutic recommendations.

A notable example of this lack of diversity is in the evaluation of therapeutics following percutaneous coronary intervention (PCI). A recent meta-analysis of 10 randomized coronary stent clinical trials reported that Black and Hispanic patients constituted only 4 and 2%, respectively, of the enrolled participants (7). However, Black patients (23.9%) and Hispanic patients (21.5%) had a higher 5-year risk for MACE when compared to White patients (18.8%) (7). The significant under representation of Black, Hispanic, and other minority participants has also been evident in clinical studies of antiplatelet therapy in patients undergoing PCI.

The standard of care in patients undergoing PCI is dual antiplatelet therapy with a P2Y_12_ inhibitor (clopidogrel, prasugrel, or ticagrelor) and aspirin to prevent major adverse cardiovascular events (MACE) such as death, stent thrombosis, myocardial infarction (MI), and stroke (8). Prasugrel and ticagrelor have shown superior efficacy compared to clopidogrel in clinical trials of acute coronary syndrome (ACS) patients in which the majority of patients underwent PCI; however, these alternative agents are more expensive and associated with higher bleeding risk and discontinuation rates compared to clopidogrel (9–11). Although clinical guidelines recommend use of prasugrel or ticagrelor over clopidogrel in ACS patients undergoing PCI, based on clinical trial results, clopidogrel remains the most widely prescribed P2Y_12_ inhibitor in clinical practice (8, 11).

Clopidogrel is a prodrug that requires bioactivation by the CYP2C19 enzyme into its active metabolite. It is well established that *CYP2C19* no function alleles result in an impaired capacity to convert clopidogrel into its active metabolite and diminished inhibition of platelet reactivity (12). Thus, clopidogrel-treated patients who carry one or two *CYP2C19* no function alleles are at higher risk of MACE after PCI (13). In contrast, prasugrel and ticagrelor clinical response is not affected by *CYP2C19* genotype (14, 15). Accumulating evidence from multiple randomized and observational studies has demonstrated that *CYP2C19* genotype guided antiplatelet therapy following PCI improves clinical outcomes (12, 16). Although use of *CYP2C19* genotype to guide antiplatelet therapy selection has not been widely adopted, an increasing number of institutions have implemented this precision medicine strategy into clinical practice (17, 18). However, most pharmacogenomic studies evaluating *CYP2C19* genotype associations with clopidogrel response and clinical outcomes of genotype-guided antiplatelet therapy to date have lacked racial and ethnic diversity.

In this review, we will (1) summarize current guideline recommendations and clinical outcome evidence related to *CYP2C19* genotype-guided antiplatelet therapy, with a particular focus on ACS/PCI patients, (2) evaluate the presence of potential racial and ethnic disparities in the major outcome studies supporting current genotype-guided antiplatelet therapy recommendations, and (3) identify remaining knowledge gaps and future research directions necessary to advance implementation of precision medicine for dual antiplatelet therapy in diverse, real-world clinical settings.

## P2Y_12_ inhibitor overview

Clopidogrel is a thienopyridine prodrug that requires hepatic biotransformation by CYP enzymes to generate an active metabolite, which irreversibly inhibits the adenosine diphosphate (ADP) P2Y_12_ receptor. Approximately 85% of clopidogrel is hydrolyzed by carboxylesterase-1, leaving 15% available for active metabolite formation by CYP2C19 and other CYP isoforms. Prasugrel is also a thienopyridine prodrug. However, in contrast to clopidogrel, prasugrel undergoes bioactivation by CYP3A4 and CYP2B6, and to a lesser extent by CYP2C19 (16). Ticagrelor, a cyclopentyl-triazolopyrimidine, is a reversible and non-competitive P2Y_12_ inhibitor that is bioactive and also metabolized by CYP3A4 into an active metabolite (16).

Overall, prasugrel and ticagrelor exhibit more predictable and consistent antiplatelet effect compared with clopidogrel (9, 10). In the Trial to Assess Improvement in Therapeutic Outcomes by Optimizing Platelet Inhibition with Prasugrel–Thrombolysis in Myocardial Infarction (TRITON–TIMI) 38 and Study of Platelet Inhibition and Patient Outcomes (PLATO) randomized clinical trials, prasugrel and ticagrelor displayed superior efficacy compared to clopidogrel in acute coronary syndrome (ACS) patients in the absence of *CYP2C19* genotyping; however, these agents were associated with increased bleeding risk (9, 10). Of note, approximately 92% of participants in the PLATO and TRITON-TIMI 38 trials were White; therefore, it remains unknown whether the clinical benefit of these agents also extend to underrepresented minority populations.

According to current clinical guidelines, prasugrel or ticagrelor is recommended over clopidogrel in ACS patients undergoing PCI based on data from these comparative trials with clopidogrel (8). Although use of prasugrel and ticagrelor has increased substantially over the past decade, clopidogrel remains the most widely prescribed P2Y_12_ inhibitor in clinical practice (8, 11). A retrospective national cohort study evaluated the prescribing patterns of P2Y_12_ inhibitors in patients who underwent PCI between 2008 and 2016 and found that approximately 74% patients filled a prescription for clopidogrel and approximately 25% of patients filled a prescription for prasugrel or ticagrelor (11). Evaluation of the demographic data from the study found that use of clopidogrel was similarly high in White patients (74%), Black patients (77%), and Hispanic patients (76%), and slightly lower in Asian patients (69%) (11). Therefore, clopidogrel remains the most common P2Y_12_ inhibitor prescribed, irrespective of race and ethnicity (11). Several more recent studies among ACS patients have reported higher use of alternative therapies in White patients compared to non-White patients (19, 20). For instance, Hispanic ethnicity was found to be independently associated with the initiation of clopidogrel compared to prasugrel or ticagrelor among ACS patients (20).

Patient demographics and socioeconomic characteristics can influence medication adherence. Overall, prasugrel and ticagrelor are associated with lower medication adherence rates when compared to patients who are prescribed clopidogrel after PCI (11), which may be related to higher rates of minor bleeding and other factors such as cost and ticagrelor-associated dyspnea and twice daily dosing. Although prasugrel is available generically, clopidogrel prescription costs remain lower. Ticagrelor remains patent restricted and has the highest costs. A study also found that non-White race and residence in lower income communities were associated with lower P2Y_12_ inhibitor adherence rates (11). Additionally, Black race, Asian race, and Hispanic ethnicity were associated with significantly lower P2Y_12_ inhibitor adherence over 6 months following PCI for ACS patients (20). Taken together, these studies illustrate that race and ethnicity are associated with P2Y_12_ inhibitor prescribing and adherence in clinical practice.

## Overview of *CYP2C19* genotype and response to P2Y_12_ inhibitors

**FIGURE 1:**
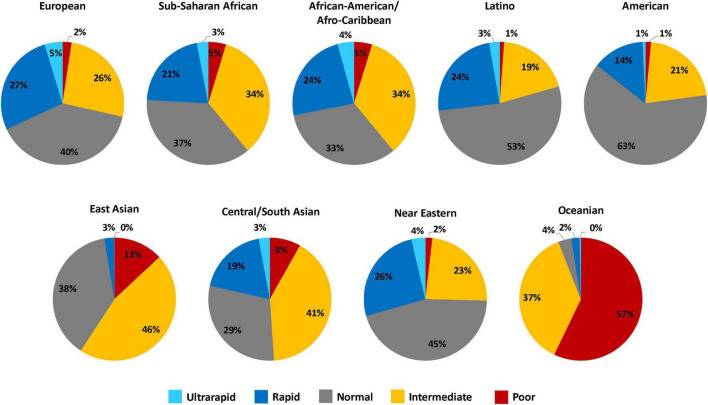
CYP2C19 metabolizer phenotype frequency estimates across diverse biogeographical groups. This figure summarizes the relative frequency estimates (in percentages) of CYP2C19 metabolizer phenotypes in the 9 distinct biogeographical groups defined by PharmGKB to annotate racial and ethnicity information about participants in pharmacogenomic studies (21). The CYP2C19 metabolizer phenotype and respective *CYP2C19* genotypes are as categorized: ultrarapid metabolizers (*17/*17), rapid metabolizers (*1/*17), normal metabolizers (*1/*1), intermediate metabolizers (e.g., *1/*2 or *2/*17), and poor metabolizers (e.g., *2/*2). Frequency data was obtained from CPIC (12).

It is well established that substantial interpatient variability in CYP2C19 metabolism can be attributed to genetic polymorphisms in *CYP2C19* (12). Three alleles account for the majority of *CYP2C19* genetic variation across populations. CYP2C19*2 (rs4244285, c861G > A) and CYP2C19*3 (rs4986893, c.636G > A) are no function alleles that result in a metabolically inactive CYP2C19 protein, and CYP2C19*17 (rs12248560, -806C > T) is an increased function allele that increases enzyme expression (12). As defined by the Clinical Pharmacogenetics Implementation Consortium (CPIC), the combination of no function and increased function alleles results in five predicted CYP2C19 activity phenotypes: ultrarapid metabolizers (UM) (*17/*17), rapid metabolizers (RM) (*1/*17), normal metabolizers (NM) (*1/*1), intermediate metabolizers (IM) (e.g., *1/*2 or *2/*17), and poor metabolizers (PM) (e.g., *2/*2) (Figure 1) (12). The frequency of *CYP2C19* polymorphisms and metabolizer phenotypes vary across different biogeographical groups used by Pharmacogenomics Knowledge Base (PharmGKB) to annotate racial and ethnicity information about participants in pharmacogenomic studies (21). Approximately 30% of European, 40% of Sub-Saharan African, 40% of African American/Afro-Caribbean, 20% of Latino, 23% of American, 60% of East Asian, 50% of Central/South Asian, 25% of Near Eastern, and 94% of Oceanian populations carry a *CYP2C19* no function allele (Figure 1). Therefore, when compared to individuals of European ancestry, CYP2C19 IMs and PMs are slightly more prevalent in individuals of African ancestry, approximately two times more common in patients of East Asian ancestry, and almost exclusively prevalent in patients of Oceanian ancestry.

It is well-established that substantial interpatient variability in platelet inhibition exists in those treated with clopidogrel, and genetic polymorphisms significantly contribute to observed variability in clopidogrel response and platelet reactivity (22, 23). CYP2C19 IMs and PMs have a significantly reduced capacity to convert clopidogrel into its active metabolite and diminished inhibition of platelet activation compared to patients who do not carry a *CYP2C19* no function allele (24). Additionally, *CYP2C19* no function allele carriers treated with clopidogrel have significantly higher rate of high on-treatment platelet reactivity (HTPR), which is associated with a higher risk of MACE (25). Multiple retrospective studies and meta-analyses have consistently shown that CYP2C19 IM and PMs treated with clopidogrel have an increased risk of MACE and stent thrombosis after PCI compared to those without a no function allele (13, 15, 24, 26). In contrast to clopidogrel, the pharmacokinetics, antiplatelet effects, and clinical effectiveness of prasugrel and ticagrelor are not affected by *CYP2C19* genotype (12). *Post hoc* genetic analyses of the TRITON-TIMI 38 and PLATO clinical trials demonstrated that *CYP2C19* genotype has no effect on outcomes after PCI among patients randomized to ticagrelor or prasugrel (14, 15, 27).

Increased risk for MACE and stent thrombosis in clopidogrel-treated IMs and PMs has been shown in prior meta-analyses of predominantly European ancestry populations (MACE: HR 1.55, 95% CI: 1.11–2.17 for IMs and HR 1.76, 95% CI: 1.24–2.50 for PMs; stent thrombosis: HR 2.81, 95% CI: 1.81–4.37 for IMs and PMs combined) (13) and East Asian ancestry populations (MACE: odds ratio [OR] 1.92, 95% CI: 1.34–2.76 for IMs and OR 3.08, 95% CI: 1.85–5.13 for PMs; stent thrombosis: OR 4.77, 95% CI: 2.84–8.01 for IMs and PMs combined) (26). Some studies have reported that carriers of the increased function CYP2C19*17 allele exhibit higher clopidogrel active metabolite formation, inhibition of platelet activation, and bleeding risk compared to non-carriers (28, 29). However, the *17 allele does not occur on the same haplotype as the *2 allele; therefore, these associations may be related to the absence of the CYP2C19*2 allele because other studies that account for the *2 allele observed no associations between CYP2C19 RMs or UM status and clopidogrel pharmacodynamics (30, 31). In addition, recent clinical outcome studies that account for the *CYP2C19* no function alleles have demonstrated no significant association between the CYP2C19*17 allele and bleeding and ischemic outcomes in clopidogrel-treated PCI patients (31–33).

The retrospective studies establishing the effects of *CYP2C19* genetic variation on clopidogrel responsiveness and outcomes after PCI have been conducted predominantly in populations of European (13) or Asian ancestry (26), and studies investigating associations with clinical outcomes in populations of African ancestry, Hispanic ethnicity, and other under-represented populations are lacking. Therefore, the association between *CYP2C19* no function alleles, clopidogrel response, and major cardiovascular outcomes remains unclear in other racial and ethnic populations because of limited data and the lack of diversity in these clinical pharmacogenomic discovery studies. The best available evidence is derived from a race stratified analysis of the Translational Research Investigating Underlying Disparities in Acute Myocardial Infarction Patients’ Health Status (TRIUMPH) cohort, a multicenter U.S. registry of acute MI patients. An *ad hoc* genetic analysis of 2,732 patients (2,062 White patients, 670 Black patients) treated with clopidogrel revealed racial differences in the association between *CYP2C19* genotype and 1-year mortality (34). The investigators observed significantly higher mortality among White patients (adjusted HR 1.70, 95% CI: 1.01–2.86, *p* = 0.046) who carried the no function CYP2C19*2 allele, when compared to non-carriers; in contrast, a *2 allele association with mortality was not observed among Black patients (adjusted HR 0.63, 95% CI: 0.28–1.41, *p* = 0.262) (34). Among Black patients, however, clopidogrel-treated carriers of the increased function CYP2C19*17 allele had a significantly higher risk of mortality and bleeding compared to CYP2C19*1 homozygous individuals; no association between the *17 allele and outcomes was observed in White patients. Given the sample size limitations, these findings should be interpreted with caution until validated in an independent cohort.

## Current guideline recommendations for genotype guided antiplatelet therapy

Clopidogrel’s prescribing information considers the association between *CYP2C19* no function alleles, clopidogrel pharmacokinetics, and diminished clinical effectiveness. In 2010, the US Food and Drug Administration (FDA) added a Boxed Warning to the clopidogrel label regarding the diminished effectiveness of clopidogrel in PMs (35). In 2016, this warning was extended to include all clopidogrel indications, and is among the strongest pharmacogenomic warnings provided by the FDA in a drug label (36). Notably, the FDA boxed warning does not require genetic testing to initiate clopidogrel therapy. Therefore, if a patient’s genotype is not known, the decision to perform *CYP2C19* testing remains at the discretion of the clinician.

Clinical practice guidelines vary regarding recommendations for *CYP2C19* genetic testing. CPIC provides guidelines on the use of *CYP2C19* genotyping test results when considering clopidogrel as an antiplatelet therapy agent; notably, these recommendations are based under the assumption that genetic tests results are available (12). The recently published 2022 CPIC guideline update for *CYP2C19*-clopidogrel recommended to avoid clopidogrel and use prasugrel or ticagrelor in CYP2C19 IM or PMs in the absence of contraindications to alternative therapy, increased the strength of the recommendation for IMs in the setting of ACS or PCI to strong, and expanded recommendations to also consider patients receiving antiplatelet therapy for neurovascular indications (12). The American College of Cardiology Foundation, American Heart Association, and the Society for Cardiovascular Angiography and Interventions (ACCF/AHA/SCAI) guidelines recommended that *CYP2C19* genetic testing may be considered in patients undergoing PCI who are at high risk for poor clinical outcomes due to inadequate platelet inhibition (Class IIB, Level of Evidence C) but recommended against routine *CYP2C19* genetic testing in all ACS patients undergoing PCI (8, 37). These recommendations have remained unchanged since 2011.

In 2019, the European Society of Cardiology (ESC) provided an updated expert consensus statement, which noted that *CYP2C19* genotyping in patients undergoing PCI with stable CAD or ACS on clopidogrel treatment may provide useful data for cardiovascular risk prediction for bleeding and ischemic events (23). However, routine genotyping to guide P2Y_12_ inhibitor treatment was not recommended because clinical trial evidence supporting the utility of these strategies was lacking. In 2020, the ECS guidelines stated that *CYP2C19* genotyping to guide dual antiplatelet therapy de-escalation (switch from prasugrel or ticagrelor to clopidogrel) in selected Non-ST-segment elevation acute coronary syndrome (NSTE-ACS) patients may be considered as an alternative to 12 months of potent platelet inhibition, especially for patients deemed unsuitable for maintained potent platelet inhibition (38).

Altogether, current clinical guidelines provide clinicians the opportunity to utilize *CYP2C19* genotyping to guide antiplatelet therapy selection after PCI in selected, high-risk patients. However, these guideline recommendations were based on evidence from clinical studies that were primarily conducted in patients of European or Asian ancestry and lacked racial and ethnic diversity, and do not directly comment on whether the evidence and recommendations should be extrapolated to underrepresented populations.

## Summary of major acute coronary syndrome/percutaneous coronary intervention clinical outcome studies that utilized *CYP2C19* genotyping

Recent studies support the use of a genotype-guided antiplatelet selection strategy in clinical practice (39). Collectively, multiple prospective randomized clinical trials (RCTs) (40–43) and observational studies (44–50) have demonstrated that *CYP2C19* genotype-guided selection of P2Y_12_ inhibitor therapy improves clinical outcomes in the setting of ACS/PCI. The major randomized and observational outcome studies that evaluated prospective *CYP2C19* genotyping in ACS/PCI patients are summarized in Table 1, and the 4 major recent studies that reported race and ethnicity data are described in greater detail below.

**TABLE 1:** Major prospective studies reporting clinical outcomes of *CYP2C19* genotype-guided antiplatelet therapy after PCI.

	Study (Sample size)	Sites and location	Race and ethnicity reported	Treatment strategy	Major findings
**Prospective genotyping (Randomized trials)**	TAILOR-PCI (*N* = 5,276) (51)	40 centers in the U.S., Canada, South Korea, and Mexico	Yes	Universal clopidogrel vs. genotyped-guided escalation strategy: clopidogrel (NMs) or ticagrelor (IM/PMs)	Among CYP2C19 IM/PMs, genotype-guided therapy (ticagrelor) exhibited a numerically lower risk of MACE compared to conventional therapy (clopidogrel) at 1 year (4.0% vs. 5.9%, HR: 0.66; 95% CI: 0.43–1.02; *p* = 0.06); however, this difference was not statistically significant.
	POPular-Genetics (*N* = 2,488) (42)	10 centers in Europe	Yes	Universal prasugrel or ticagrelor vs. genotype-guided de-escalation strategy: clopidogrel (NMs) or prasugrel/ticagrelor (IM/PMs)	*CYP2C19* genotype guided therapy was non-inferior to universal prasugrel or ticagrelor for the risk of MACE or major bleeding (5.1% vs. 5.9%, absolute difference: -0.7%; 95% CI: -2.0 to 0.7; *p* < 0.001 for non-inferiority).
	PHARMCLO (*N* = 888)* (41)	12 centers in Italy	No	Standard-of-care vs. genotype-guided escalation strategy with treatment at physician discretion	*CYP2C19* genotype guided therapy reduced the risk of MACE or major bleeding compared to standard of care at 1 year (15.9% vs. 25.9%; HR: 0.58; 95% CI: 0.43–0.78; *p* < 0.001).
	IAC-PCI (*N* = 600) (40)	Single center in China	No	Universal clopidogrel vs. genotype-guided escalation strategy: clopidogrel (NMs), high dose clopidogrel (IMs), or high dose clopidogrel + cilostazol (PMs)	*CYP2C19 g*enotype guided therapy decreased risk of MACE compared to universal clopidogrel at 180 days (2.7% vs. 9.0%; *p* = 0.001).
**Prospective genotyping (Observational trials)**	IGNITE (*N* = 3,342) (50)	9 centers in the U.S.	Yes	Genotype-guided therapy (prasugrel/ticagrelor recommended in IM/PMs) with treatment decision at physician discretion	Among CYP2C19 IM/PMs, patients prescribed alternative therapy had significantly lower risk of MACE over 1 year after PCI compared to those prescribed clopidogrel (adjusted HR: 0.56; 95% CI: 0.39–0.82; *p* = 0.002). In non-IM/PMs, no difference observed (18.1 vs. 19.9 per 100-pt years; adjusted HR: 1.08; 95% CI: 0.72–1.62; *p* = 0.715).
	GIANT (*N* = 1,445) (48)	57 centers in France	No	Genotype-guided therapy (prasugrel recommended in PMs and either prasugrel or high dose clopidogrel recommended in IMs) with treatment decision at physician discretion	Compared to CYP2C19 non-IM/PMs prescribed clopidogrel, MACE rates were significantly higher in IM/PMs prescribed clopidogrel (3.04% vs. 15.6%; *p* < 0.05) but not significantly different in IM/PMs prescribed alternative therapy (3.04% vs. 3.31%; *p* = 0.82).
	PHARM-ACS (N = 1,361) (49)	Single center in China	Yes	Genotype-guided therapy (ticagrelor recommended in IM/PMs) with treatment decision at physician discretion	CYP2C19 IM/PMs prescribed clopidogrel experienced a significant higher risk of MACE compared to those prescribed ticagrelor (7.8% vs. 4.0%; adjusted HR: 2.14; 95% CI: 1.30–3.52). In non-IM/PMs, no significant difference was observed across the clopidogrel vs. ticagrelor groups (5.8% vs. 4.3%; adjusted HR:1.06; 95% CI: 0.59–1.90).
	Sánchez-Ramos et al. (*N* = 719) (45)	Single center in Spain	No	Conventional therapy^ vs. genotype-guided clopidogrel (NMs) or prasugrel/ticagrelor (IM/PMs)	*CYP2C19* genotype guided therapy was associated with a lower risk of MACE compared to historical controls on conventional therapy at 1 year (10.1% vs. 14.1%; HR: 0.63; 95% CI: 0.41–0.97; *p* = 0.037).
	Shen et al. (*N* = 628) (46)	Single center in China	No	Universal clopidogrel vs. genotype-guided clopidogrel (NMs), high dose clopidogrel (IMs), or ticagrelor (PMs)	*CYP2C19* genotype guided therapy was associated with a lower risk of MACE compared to universal clopidogrel at 1 year (4.2% vs. 9.4%; *p* = 0.010).

MACE, major adverse cardiovascular events (the definition in each study was slightly different, and is described in the text); MI, myocardial infarction; BARC, Bleeding Academic Research Consortium; TIMI, Thrombolysis in Myocardial Infarction; GUSTO, Global Use of Strategies to Open Occluded Arteries; NMs, normal metabolizers; IMs, intermediate metabolizers; PMs, poor metabolizers.

*The study was discontinued prematurely due to lack of genotyping instrument certification, and only enrolled approximately 25% of the pre-specified sample size.

^Historical unguided control group (N = 402) in which a majority patients received clopidogrel and 7% received prasugrel.

These outcome studies have informed three recent major meta-analyses. A meta-analysis of 15,949 patients (98% ACS, 77% undergoing PCI) from 7 randomized trials reported that treatment with prasugrel or ticagrelor reduced major ischemic events compared to clopidogrel in CYP2C19 IMs and PMs (RR 0.70, 95% CI: 0.59–0.83), whereas no difference was observed in patients who were non-carriers of no function alleles (RR 1.0, 95% CI: 0.80–1.25) (51). A significant genotype-treatment interaction (*p* = 0.013) was reported, which suggests that the reduction of ischemic events by prasugrel or ticagrelor, in comparison with clopidogrel, was driven in large part by *CYP2C19* genotype and the magnitude of the benefit was greatest in CYP2C19 IMs and PMs (51). An additional meta-analysis that included 20,743 patients from 14 studies reported that genotyped-guided antiplatelet therapy selection significantly reduced the risk of MACE compared with standard non-guided antiplatelet therapy (RR 0.78, 95% CI: 0.63–0.95, *p* = 0.015) (52). A network meta-analysis of 61,898 ACS patients from 15 randomized trials also reported that a guided approach of P2Y_12_ antiplatelet therapy selection was associated with reduced MACE [incidence rate ratios (IRR) 0.80, 95% CI: 0.65–0.98] without a significant increase in all bleeding (IRR 1.22, 95% CI: 0.96–1.55) compared to routine selection of prasugrel or ticagrelor without genotyping (53). Collectively, these meta-analyses support the use of genetic testing to optimize the choice of agent in patients undergoing PCI. However, the race and ethnicity composition of the meta-analysis populations were not reported.

## Prospective genotyping (Randomized trials)

### TAILOR-PCI

The Tailored Antiplatelet Initiation to Lessen Outcomes due to Decreased Clopidogrel Response After Percutaneous Coronary Intervention (TAILOR PCI) was a randomized, open-label, superiority, multicenter trial of *CYP2C19* genotype-guided antiplatelet therapy conducted in 5,276 patients (43). The study population consisted of 66.4% White, 2.4% Black of African-American, 22.5% East Asian, 4.5% South Asian, and 2.8% Hispanic or Latino patients; 4.3% reported another race or were of unknown race (54). Patients undergoing PCI for an ACS or non-ACS indication were randomized within 72 h after PCI to conventional therapy (universal clopidogrel without initial genetic testing) or to genotype-guided therapy [ticagrelor in *CYP2C19* no function allele carriers (IMs or PMs), and standard-dose clopidogrel in non-carriers]. At the end of the trial, patients in the conventional therapy group underwent *CYP2C19* genotyping, and the primary analysis compared outcomes in *CYP2C19* no function allele carriers across the genotype-guided group (*n* = 903) and the universal clopidogrel group (*n* = 946). The primary outcome was a composite of cardiovascular death, MI, stroke, stent thrombosis, and severe recurrent ischemia at 12 months. Overall, CYP2C19 IM/PMs treated with ticagrelor in the genotyped-guided group had a numerically lower rate of the primary outcome compared to CYP2C19 IM/PMs receiving clopidogrel in the conventional therapy group (4.0% vs. 5.9%; HR: 0.66; 95% CI: 0.43–1.02; *p* = 0.06). However, the event rate was lower than anticipated and the difference was not statistically significant. In a *post hoc* analysis, IM/PMs receiving ticagrelor had a lower risk of ischemic events at 90 days compared to clopidogrel (HR 0.21; 95% CI: 0.08–0.54; *p* = 0.001). There was no significant difference in the primary safety end point of major or minor bleeding rates across groups (HR 1.22; 95% CI: 0.60–2.51; *p* = 0.58).

### POPular-genetics

The *CYP2C19* Genotype-Guided Antiplatelet Therapy in ST-Segment Elevation Myocardial Infarction Patients—Patient Outcome after Primary PCI (POPular Genetics) trial was a randomized, multicenter, open-label, non-inferiority trial conducted in 2,488 ST segment elevation MI (STEMI) patients undergoing PCI (42). The study population consisted of 94.3% European or White, 0.2% Black, 2.8% Asian, and 1.0% Hispanic or Latino patients; < 2% of participants did not report race or ethnicity. POPular Genetics evaluated whether a *CYP2C19* genotype-guided antiplatelet therapy de-escalation strategy reduced bleeding risk without increasing thrombotic risk compared to conventional therapy with ticagrelor or prasugrel. The study randomized patients during or within 48 h after PCI to conventional treatment (universal ticagrelor or prasugrel without genetic testing) or genotype-guided therapy (prasugrel or ticagrelor in *CYP2C19* no function allele carriers [IMs and PMs], and standard-dose clopidogrel in non-carriers). Overall, the genotype-guided strategy was non-inferior to universal ticagrelor or prasugrel in occurrence of the primary composite outcome of death, MI, stent thrombosis, stroke, or major bleeding events at 12 months (5.1% vs. 5.9%; absolute difference: -0.7%; 95% CI: -2.0 to 0.7; *p* < 0.001 for non-inferiority). Additionally, the genotype guided de-escalation strategy significantly reduced the co-primary outcome of major or minor bleeding rates (9.8% vs. 12.5%; HR: 0.78; 95% CI, 0.61–0.98; *p* = 0.04), which was driven by a lower incidence of minor bleeding because no significant difference in major bleeding events were observed.

## Prospective genotyping (observational studies)

### Implementing genomics in practice

A multicenter pragmatic study conducted by U.S. early adopter institutions in the Implementing Genomics in Practice (IGNITE) Network, examined clinical outcomes following clinical implementation of *CYP2C19* genotype-guided antiplatelet therapy after PCI in a real-world clinical setting (47, 50). As part of the clinical implementation at each site, prasugrel or ticagrelor was recommended in CYP2C19 IMs and PMs in the absence of contraindications; however, the ultimate prescribing decision was left to the clinician. The initial analysis conducted in 1,815 patients across 7 centers demonstrated that CYP2C19 IM/PMs prescribed clopidogrel experienced significantly higher MACE rates over 12 months compared to IM/PMs prescribed alternative therapy (adjusted HR: 2.26; 95% CI: 1.18–4.32; *p* = 0.013) (47). A more recent analysis was conducted in an expanded cohort of 3,342 patients across 9 centers, and is described in greater detail below (50). The study population demographics of the initial and expanded cohort were comparable, and consisted of approximately 70% European or White, 20% African American or Black, 1% Asian, and 4% Hispanic or Latino patients; 1% of patients reported another race or multiple races, and 3% did not have race or ethnicity information available in the electronic health record.

The primary outcome assessed in the recent expanded cohort analysis was major atherothrombotic events, defined as a composite of death, MI, ischemic stroke, stent thrombosis, or hospitalization for unstable angina, over 12 months after PCI (50). Major atherothrombotic event rates were significantly lower in CYP2C19 IM/PMs prescribed alternative therapy vs. those who were prescribed clopidogrel (17.1 vs. 34.4 per 100 patient-years, respectively; adjusted HR: 0.56; 95% CI: 0.39–0.82; *p* = 0.002); however, no significant difference was observed across alternative therapy and clopidogrel groups in patients without a no function allele (18.1 vs. 19.9 per 100 patient-years, respectively; adjusted HR: 1.08; 95% CI: 0.72–1.62; *p* = 0.715). The observed differences in IM/PMs were most pronounced in ACS patients undergoing PCI (adjusted HR: 0.49; 95% CI: 0.32–0.76; *p* = 0.001), whereas use of clopidogrel or alternative therapy were similarly effective in ACS patients without a no function allele (adjusted HR: 1.05; 95% CI: 0.67–1.66; *p* = 0.834). There was no difference in major bleeding rates between the alternative therapy group vs. clopidogrel group in either IM/PMs (adjusted HR; 1.15; 95% CI: 0.60–2.20; *p* = 0.685), or non-IM/PMs (adjusted HR: 1.30; 95% CI: 0.71–2.38; *p* = 0.397). A separate analysis from this population focused on the increased function CYP2C19*17 allele demonstrated that clopidogrel-treated RMs or UMs exhibited no difference in atherothrombotic (adjusted HR: 0.97; 95% CI: 0.73–1.29; *p* = 0.808) or bleeding events (adjusted HR: 1.34; 95% CI: 0.83–2.17; *p* = 0.224) compared to clopidogrel-treated NMs (33).

### PHARM-ACS

The PHARMacotherapy and long-term clinical outcomes in patients with ACS after PCI (PHARM-ACS) study was a single-center observational cohort study conducted in China that evaluated the effect of *CYP2C19* genotype-guided antiplatelet therapy on clinical outcomes in 1,361 patients with ACS after PCI (49). Approximately 98% of the participants identified as Han nationality, and 60.7% carried at least one no function allele. Ticagrelor was recommend in CYP2C19 IMs and PMs, but the ultimate prescribing decision was left to clinician’s discretion. The primary endpoint was a composite of death, stent thrombosis, stroke, MI, and any urgent coronary revascularization within 1 year after PCI. Consistent with the IGNITE study results, use of clopidogrel in IM/PMs was associated with a significantly higher risk of MACE compared to IM/PMs prescribed ticagrelor (adjusted HR: 2.14; 95% CI: 1.30–3.52), and no differences in MACE risk were observed across groups in non-IM/PMs (adjusted HR: 1.06; 95% CI: 0.59–1.90). There was also no significant difference in bleeding events across groups.

## Racial and ethnic disparities in major *CYP2C19* genotyping outcome studies in acute coronary syndrome/percutaneous coronary intervention patients that support current genotype-guided antiplatelet therapy recommendations

**FIGURE 2:**
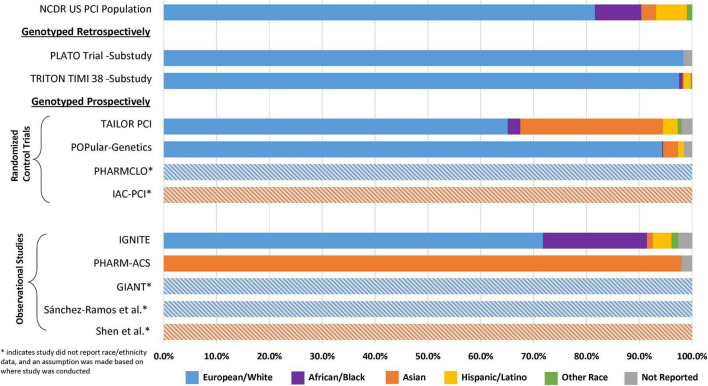
Reported race and ethnicity data from selected major clinical outcome trials utilizing clopidogrel and in which *CYP2C19* status was reported. This figure compares the relative percent distribution of reported race and ethnicity from study participants included major retrospective and prospective clinical outcome studies of *CYP2C19* genotype-guided antiplatelet therapy. *Studies with the gray hatched bars did not report race and ethnicity data, and an assumption about the population demographics was made based on the study site locations described in Table 1 (Europe or China). For reference, the demographic characteristics of each study were compared to data obtained from the National Cardiovascular Data Registry (NCDR): the race and ethnicity distribution of patients who underwent PCI in the U.S. was 86.5% White or European, 8.8% Black or African American, 2.8% Asian, 0.7% Native American, 0.3% Pacific Islander, and 5.8% Hispanic or Latino ethnicity (55).

In order to assess the presence of disparities in the major clinical outcome studies supporting current *CYP2C19* genotype-guided antiplatelet therapy recommendations, reported demographic data from 11 major clinical outcome studies of *CYP2C19* genotype guided antiplatelet therapy in ACS/PCI patients were summarized and compared (Figure 2 and Supplementary Table 1). For reference, the demographic characteristics were compared to a national database derived from 667,424 patient records across 1,612 U.S. centers obtained from the National Cardiovascular Data Registry (NCDR). In 2014, the race and ethnicity distribution of patients who underwent PCI in the U.S. was 86.5% White or European, 8.8% Black or African American, 2.8% Asian, 0.7% Native American, 0.3% Pacific Islander, and 5.8% Hispanic or Latino ethnicity (55).

**FIGURE 3:**
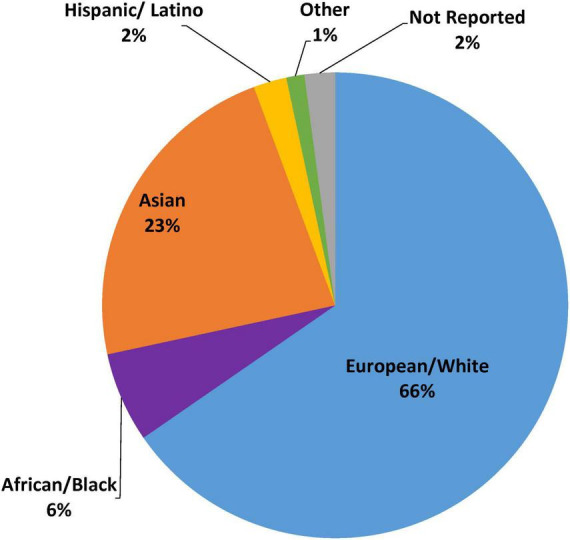
Representation of race and ethnicity data from selected *CYP2C19* outcome studies. This figure summarizes race and ethnicity distribution of individuals included in major prospective clinical outcome studies of *CYP2C19* genotype guided antiplatelet therapy (as summarized in Supplementary Table 1). Pie graph displays aggregated percent race and ethnicity composition from the *N* = 12,467 patients enrolled in the 4 studies with reported race and ethnicity data (TAILOR PCI, POPular Genetics, IGNITE, PHARM-ACS).

The retrospective genetic analyses of the TRITON-TIMI 38 and PLATO RCTs, which established that *CYP2C19* no function alleles significantly diminish clopidogrel but not ticagrelor or prasugrel clinical effectiveness, were conducted almost exclusively in patients of European ancestry (∼98%) (14, 15, 24, 27). Of the 9 major randomized and observational outcome studies that conducted prospective *CYP2C19* genotyping in ACS/PCI patients summarized in Table 1, 4 studies (TAILOR-PCI, POPular-Genetics, IGNITE, PHARM-ACS) reported participant-level data on the race and ethnicity of the study participants (42, 43, 49, 50). Of these 4 studies, 3 reported White or European representation, 3 reported Black or African American representation, 3 reported Hispanic or Latino representation, 4 reported Asian representation, and 3 reported representation of other races. Aggregation of race and ethnicity data across the 12,467 patients included in these 4 studies (Figure 3) demonstrated that the majority of the study participants identified as European or White (66%). There was also strong representation of Asian patients in these studies (23%), which predominantly included patients of East Asian ancestry. Moreover, of the 5 studies with unreported race and ethnicity data (Figure 2), PHARMCLO (multiple centers in Italy), GIANT (multiple centers in France), and Sánchez-Ramos et al. (single center in Spain) were conducted exclusively in Europe, and IAC-PCI and Shen et al. were conducted at single centers in China (40, 41, 45, 46, 48). Although the self-identified race and ethnicity of the study participants were not reported, the study locations suggest that the study participants predominantly represented European and East Asian ancestry, respectively. In contrast, only about 6% of participants in these 4 studies identified as Black or African American and 2% identified as Hispanic or Latino (Figure 3), which is lower than the U.S. PCI population reported by NCDR. Among the patients that identified as another race in these studies (1%), the proportion of Native American, Pacific Islander, and multiracial patients were unclear and thus these populations were also mostly likely underrepresented.

Although there is accumulating evidence supporting the clinical utility of *CYP2C19* genotype-guided antiplatelet therapy selection in the setting of ACS/PCI, our review of the evidence demonstrates a collective lack of racial and ethnic diversity in the major clinical outcome studies supporting recent guideline recommendations and has identified important evidence gaps regarding the effectiveness of this precision medicine strategy in underrepresented populations. There remain limited clinical outcome data in patient populations beyond those of European and East Asian ancestry. Therefore, the benefits and risks of this precision medicine strategy in Black, Hispanic, and other underrepresented populations remain unclear. Future outcome studies in diverse real-world clinical settings are critical to address racial and ethnic disparities in the evidence base and equitably evaluate clinical utility of *CYP2C19* genotype-guided antiplatelet therapy in ACS/PCI patients.

## Racial and ethnic disparities in discovery studies that identify and validate genetic predictors of clopidogrel response

It is well established that genomic and pharmacogenomic discovery studies have lacked racial and ethnic diversity and been predominantly conducted in populations of European ancestry (56–58). Evaluation of the distribution of ancestry categories within genome-wide association studies (GWAS) from 2005 to 2016 in the NHGRI-EBI GWAS Catalog revealed that European ancestry individuals have represented the overwhelming majority of participants in genetic discovery studies (78%) (59). The GWAS studies compromised Asian individuals (11%), with East Asian ancestry (9%) accounting for most and South/Central Asian ancestry (2%) less well represented, followed by African ancestry (2%), Hispanic or Latin American individuals (1%), all other populations (< 1%), and reported samples where the ancestry category could not be specified (6%) (59, 60). It is well-established that African populations have the greatest genetic diversity and largest number of population-specific alleles (61, 62). African ancestry populations have contributed to a disproportionately higher number of genome-wide significant associations (7%) when compared to the representation in GWAS studies (∼2%); the opposite trend exists in individuals of European ancestry (54% of associations with 78% of participants) (59, 60). Therefore, failure to enhance ancestral diversity in genomic research studies will augment health disparities in underrepresented populations (58). Enhancing ancestral diversity in genomic discovery studies offers enormous potential to advance the discovery of genetic predictors of disease risk and drug response and optimize the development of precision medicine interventions that can more equitably improve outcomes in individual patients.

This problem is evident when specifically evaluating the evidence underlying the discovery of genetic factors associated with inter-patient variability in clopidogrel response. A GWAS in 429 healthy Amish volunteers of European ancestry determined that the CYP2C19*2 no function allele accounted for approximately 12% of the variation in clopidogrel on-treatment platelet reactivity and was the only significant genome-wide association (63). A subsequent GWAS in 513 Amish volunteers from the same study population demonstrated that CYP2C19*2 exhibited the strongest association with clopidogrel active metabolite levels (64). The largest GWAS of clopidogrel response was conducted in 2,750 ACS/PCI patients of European ancestry by the International Clopidogrel Pharmacogenomics Consortium and demonstrated that CYP2C19*2 was the strongest determinant of clopidogrel on-treatment platelet reactivity (65). A GWAS conducted in 115 Chinese patients with CAD did not identify significant genome-wide associations with clopidogrel inhibition of platelet reactivity or active metabolite levels but was limited by sample size. In this study, CYP2C19*2 accounted for approximately 11 and 16% of the variability in clopidogrel on-treatment platelet reactivity and active metabolite plasma concentrations, respectively (66). While these studies in European and East Asian ancestry populations identified other potential genetic variants that may contribute to variation in clopidogrel response (31, 63–66), they collectively demonstrate that *CYP2C19* no function alleles are the strongest genetic determinant of clopidogrel response and associated with clopidogrel clinical effectiveness in European and East Asian ACS/PCI patients (13, 26). Interestingly, East Asian populations are less likely to experience thromboembolic and ischemic complications but more likely to experience bleeding complications compared to European populations, which is known as the “East Asian Paradox” (67). It remains unclear whether genetic determinants of platelet function or antiplatelet drug effects underlie this effect, and thus additional genetic discovery research beyond *CYP2C19* is needed.

Rigorous studies seeking to identify genetic predictors of clopidogrel response in patients of African ancestry, Hispanic ethnicity, and other underrepresented populations have been lacking. A notable exception is a recent study conducted in an admixed population of 474 Caribbean Hispanic ACS/PCI patients treated with clopidogrel across multiple sites in Puerto Rico (68). The average European, Native American, and African ancestry genomic proportions in the study population were 70, 11, and 19%, respectively. The study observed that the CYP2C19*2 allele exhibited the strongest genetic association with high on-clopidogrel platelet reactivity. Moreover, genetic variants in *PON1*, *ABCB1*, and *PEAR1*, which have demonstrated inconsistent associations within European populations, were also associated with clopidogrel response. Notably, African ancestry was a significant independent predictor of clopidogrel response and an interaction between African ancestry and the *PEAR1* variant was observed. Overall, approximately 19% of the variability in clopidogrel response was attributed to independent genetic and clinical factors, with CYP2C19*2 accounting for approximately 7% of the variability in this population (68). Together, these important and novel results demonstrated that *CYP2C19* no function alleles are associated with reduced clopidogrel response in a diverse population of Caribbean Hispanic patients and suggest that the effect size of the CYP2C19*2 allele may be smaller compared to White populations, other genetic variants and ancestry may contribute to variation in clopidogrel response independent of *CYP2C19*, and these effects may be augmented in patients of African ancestry.

The relative contribution of *CYP2C19* no function alleles and other genetic variants to inter-patient variation in clopidogrel response in African ancestry populations has not been rigorously investigated to date. The clinical relevance of such studies is underscored by prior studies demonstrating that Black patients treated with clopidogrel undergoing PCI have a higher prevalence of HTPR compared to White patients (56% vs. 35%, respectively, *P* = 0.003) (69). As described above, an analysis of *CYP2C1*9 variants and outcomes in 670 Black clopidogrel-treated acute MI patients revealed that the CYP2C19*2 no function allele was not associated with higher risk of adverse cardiovascular outcomes and mortality, whereas the CYP2C19*17 increased function allele was associated with higher risk of bleeding and mortality (34). Together, this limited evidence demonstrates that Black patients are at a higher risk of clopidogrel non-response, and suggests that unique genes and alleles beyond CYP2C19*2 are likely associated with clopidogrel response and effectiveness in Black populations. Therefore, a GWAS of clopidogrel response in patients of African ancestry is essential. To address this gap in precision medicine, the African American Cardiovascular Pharmacogenetic Consortium (ACCOuNT) was formed to discover novel genetic variants in African Americans related to clinically actionable cardiovascular phenotypes, which will include evaluation of clopidogrel clinical responsiveness (70).

Discovery pharmacogenomic studies in African ancestry and other underrepresented populations are needed to fully elucidate the presence and magnitude of *CYP2C19* and other genetic effects on clopidogrel clinical effectiveness, which may differ from prior studies conducted in predominantly European and East Asian populations. Discovery genetics studies across diverse populations are essential to ensure that genotype-guided approaches evaluated in clinical trials and implemented into clinical practice include the most informative and relevant alleles.

## Emerging studies evaluating *CYP2C19* genotype guided antiplatelet therapy in stroke patients

Clinical guidelines also recommend antiplatelet therapy for the treatment of acute ischemic stroke and the secondary prevention of ischemic stroke (71). Multiple RCTs have shown that short term (21–90 days) use of dual antiplatelet therapy with aspirin and clopidogrel reduces stroke recurrence in patients with acute ischemic stroke or transient ischemic attack (TIA) (72–74). Therefore, clopidogrel is commonly prescribed when a P2Y_12_ inhibitor is clinically indicated for the treatment or prevention of ischemic stroke (75).

A meta-analysis of 15 studies demonstrated a significant association between *CYP2C19* no function alleles and clinical outcomes in 4,762 clopidogrel-treated patients with stroke or TIA (76). The study population consisted of East Asian (85%), European (8%), African (2%), and other (5%) ancestry patients. *CYP2C19* no function allele carriers receiving clopidogrel had a significantly higher risk of stroke (RR: 1.92, 95% CI: 1.57–2.35) and major vascular events (RR: 1.51, 95% CI: 1.10–2.06) compared to non-carriers (76). A race-stratified subgroup analysis observed a significant increased risk of stroke in *CYP2C19* no function allele carriers of Asian ancestry (RR 1.93; 95% CI: 1.55–2.39; *P* < 0.001) and European ancestry (RR 2.46; 95% CI: 1.06–5.72; *p* = 0.04); however, the association was not statistically significant among the limited sample of African ancestry (*n* = 97) patients (RR 1.74; 95% CI: 0.63–4.79; *p* = 0.28). Additional studies in more diverse populations are needed to elucidate the presence and magnitude of *CYP2C19* genotype associations with clopidogrel clinical effectiveness beyond populations East Asian ancestry.

Emerging prospective evidence supports the use of a *CYP2C19* genotype-guided antiplatelet strategy in stroke patients. The Ticagrelor vs. Clopidogrel in CYP2C19 Loss-of-Function Carriers with Stroke or TIA (CHANCE-2) trial was a multicenter, double-blinded, placebo-controlled, randomized control, superiority trial conducted across 202 centers in China (77). The study examined whether ticagrelor plus aspirin was superior to clopidogrel plus aspirin in 6,412 patients with minor ischemic stroke or TIA who were *CYP2C19* no function allele carriers. The primary efficacy outcome was new ischemic or hemorrhagic stroke at 90 days, which occurred in 6.0% of CYP2C19 IM/PMs in the ticagrelor group and 7.6% of IM/PMs in the clopidogrel group (HR 0.77; 95% CI: 0.64–0.94; *p* = 0.008). The incidence of a major vascular event, defined as the composite of ischemic stroke, hemorrhagic stroke, TIA, MI, or cardiovascular death, was also significantly reduced in the ticagrelor group (7.2% vs. 9.2%, respectively; HR 0.77; 95% CI: 0.65–0.92). Moderate or severe bleeding occurred at 0.3% of patients in both groups (HR 0.82; 95% CI, 0.34–1.98; *p* = 0.66); however, the incidence of any bleeding was higher in the ticagrelor compared to clopidogrel group (5.3% vs. 2.5%, respectively; HR 2.18; 95% CI: 1.66–2.85). These results illustrate the clinical utility of a *CYP2C19* genotype guided strategy in the setting of acute stroke.

Black and Hispanic patients have a higher prevalence of risk factors for stroke and a higher prevalence of stroke events compared to non-Hispanic White patients (1, 78), but have been underrepresented in prior neurovascular disease studies of clopidogrel pharmacogenomics. Therefore, outcome studies evaluating the clinical impact of a genotype guided strategy in acute stroke or TIA patients need to include more diverse populations to appropriate determine the factors that influence the stroke differences among these populations underrepresented in the studies to date. In addition, outcome studies in diverse populations of patients with other neurovascular indications for clopidogrel, including neuro-interventional procedures such as carotid artery stenting and intracranial aneurysm repair, are lacking and needed.

## Increased diversity in clinical trial participation and reporting

Federal efforts and policies from the National Institute of Health (NIH) and the U.S. Food and Drug Administration (FDA) have promoted diverse clinical trial representation over time. The NIH Inclusion Policy required the inclusion of women and individuals from underrepresented minority populations in clinical research studies to enhance generalizability of findings to the patient populations being treated and enable valid subgroup analyses that evaluate outcome differences stratified by sex and race/ethnicity (79). In 2017, an amendment to the NIH Inclusion Policy required that NIH-defined Phase 3 clinical trials submit sex, race, and ethnicity data to the ClinicalTrials.gov registry (79). Recently in April 2022, the U.S. FDA issued a new draft guidance to enhance inclusion of underrepresented racial and ethnic populations in clinical trials (80). However, FDA guidance documents are recommendations that are not legally enforceable mandates. Therefore, there are major challenges to ensure that these initiatives are translated into clinical practice. As highlighted in our analysis, comprehensive reporting of race and ethnicity data in clinical trials and observational precision medicine studies (including studies not registered with FDA or funded by NIH) is necessary first step to evaluate the presence of potential disparities in in the evidence base.

Despite having a greater burden of cardiovascular disease (3, 81, 82), racial and ethnic minorities, specifically Black and Hispanic individuals, are frequently underrepresented in cardiovascular clinical research (83, 84). As described herein, this disparity also is evident in pharmacogenomics discovery and outcomes research. Underrepresented groups may often face significant barriers to clinical trial participation, including systemic racism, mistrust of the clinical research system, transportation conflicts, logistical and financial constraints, and lack of awareness and access to research information (85, 86). Strategies proposed by the Heart Failure Collaboratory to improve clinical trial enrollment of underrepresented populations include methodical research study design and site selection, diversification of research leadership and staff, review of eligibility criteria, and increased patient, institution, and community engagement (86). These strategies to improve diversity in heart failure clinical trials could be applied to clinical trials and observational studies that evaluate precision medicine strategies such as genotype-guided antiplatelet therapy. In addition, real-world studies have become increasingly important to evaluate treatment effectiveness in clinical practice. Compared to randomized clinical trials, real-world effectiveness studies are often compromised of diverse patient populations (87). Therefore, research in real-world clinical settings, such as the IGNITE Network (88), offer the potential to investigate and advance genomics discovery and implementation research into underrepresented populations.

## Summary and conclusion

Accumulating evidence from multiple randomized and observational clinical studies have demonstrated that using *CYP2C19* genotype to guide selection of antiplatelet therapy improves or is associated with improved clinical outcomes in patients with cardiovascular and neurovascular disease. This evidence has led to increased utilization of *CYP2C19* genotype-guided antiplatelet therapy in clinical practice. However, our review and analysis of major antiplatelet pharmacogenomic discovery and outcome studies revealed that these studies have lacked racial and ethnic diversity. There remain limited outcome data and major gaps in evidence regarding the effectiveness and utility of this precision medicine strategy in underrepresented minority patient populations. Additional discovery and outcomes studies that include more diverse patient populations are needed.

Although RCTs have increased rigor and decreased bias compared to observational outcome studies, RCTs of genotype guided antiplatelet therapy have not adequately represented the diversity of patient demographics within the ACS/PCI population. This is concerning particularly given the higher prevalence of cardiovascular disease and increased risk of MACE following ACS and PCI among Black and Hispanic compared to White populations. Although there are certain limitations, observational studies and pragmatic clinical trials conducted in real-world settings are more representative of the diversity of the patient population and can be used as a solution to bridge these gaps. This is evident in the diversity of the patient population included in the outcome studies conducted by the IGNITE Pharmacogenetics Working group (47, 50). In order to rigorously and equitably evaluate clinical utility, additional outcome studies of genotype-guided antiplatelet therapy conducted in diverse real-world ACS/PCI and neurovascular disease patient populations that target enrollment of key underrepresented groups should be pursued. Additional studies evaluating the clinical utility of risk stratification tools that integrate clinical and genetic factors, such as the ABCD-GENE score, in diverse patient population are warranted (89, 90).

*CYP2C19* no function alleles are common among individuals across various ancestries and certain non-European populations have higher prevalence of CYP2C19 IMs and PMs (Figure 1). Therefore, the adverse consequences of prescribing clopidogrel without genotype information is likely magnified in these populations. Most notably, Bristol-Myers Squibb Co., and Sanofi were ordered to pay the state of Hawaii more than $834 million in civil penalties for misleading marketing and failure to disclose the possibility of decreased effectiveness and diminished clopidogrel response of individuals of Asian or Pacific-Island descent (91, 92). Furthermore, because minority populations have been underrepresented in clopidogrel pharmacogenomics discovery studies, the presence and magnitude of effect of *CYP2C19* no function alleles on antiplatelet effects and MACE risk in Black, Hispanic, and other underrepresented minority populations (e.g., Native American, Pacific Islander) remains unclear. It is possible that the effect size of these associations varies across populations and genotypes beyond *CYP2C19* could be important in non-European and non-East Asian populations (68, 93). Therefore, in the absence of outcome evidence, it may not be appropriate to assume effectiveness and generalize clinical recommendations for *CYP2C19* genotype-guided antiplatelet therapy in populations that are underrepresented or excluded from these studies.

Although *CYP2C19*-clopidogrel is among the most rigorously evaluated pharmacogenomic interventions studied to date, significant racial and ethnic disparities in the evidence base remain. The conduct of discovery genetics and outcomes studies across diverse populations are essential to ensure that genotype-guided approaches used in clinical practice include the most informative and relevant alleles and improve health outcomes. In order to realize the full health benefits of genomic medicine, equitable access and inclusion of underrepresented groups is essential in research studies that seek to discover genomic predictors of disease risks, drug response, and to evaluate the clinical benefits of genomic and pharmacogenomic interventions on health outcomes.

## Author contributions

AN and CL conducted data analysis and wrote the manuscript. All authors contributed to the article and approved the submitted version.

## Abbreviations

PCI: percutaneous coronary intervention
MACE: major adverse cardiovascular events
ACS: acute coronary syndrome
CPIC: Clinical Pharmacogenetics Implementation Consortium
IM: Intermediate metabolizer
PM: poor metabolizer
RM: rapid metabolizer
UM: ultrarapid metabolizer
CDER: Center for Drug Evaluation and Research
ADP: adenosine diphosphate
STEMI: ST segment elevation myocardial infarction.
