# Supplementary material for: Evaluation of race and ethnicity disparities in outcome studies of CYP2C19 genotype-guided antiplatelet therapy
Source: Front Cardiovasc Med. 2022 Aug 23;9:991646. doi: 10.3389/fcvm.2022.991646 (PMC9445150; doi:10.3389/fcvm.2022.991646)
Supplement: Supplementary file 1 [file Table_1.pdf]

Supplemental Table 1. Summary of reported demographic characteristics from major retrospective and prospective studies reporting clinical outcomes of *CYP2C19* genotype-guided antiplatelet therapy after PCI

		Race / Ethnicity					
	**Study**	**European / White N (%)**	**African / Black N (%)**	**Asian N (%)**	**Hispanic / Latino N (%)**	**Other Race* N (%)**	**Not Reported or Unknown N (%)**
**Retrospective Genotyping (Sub-studies of major clinical trials)**	PLATO sub-study (N=10,285) (Wallentin et al., 2010)	10,115 (98.3%)	-	-	-	-	170 (1.7%)
	TRITON-TIMI 38 (N= 2,943) (SORICH et al., 2010)	2,871 (97.6%)	19 (0.6%)	9 (0.3%)	37 (1.3%)	7 (0.2%)	0 (0%)
**Prospective Genotyping (Randomized Trials)**	TAILOR-PCI (N= 5,276) (Pereira et al., 2021)	3,504 (66.5%)	124 (2.4%)	1,423^ (27.0%)	148 (2.8%)	115 (2.2%)	110 (2.1%)
	POPular-Genetics (N= 2,488) (Claassens et al., 2019)	2347 (94.3%)	5 (0.2%)	70 (2.8%)	25 (1.1%)	-	41 (1.5%)
	PHARMCLO (N= 888) (Notarangelo et al., 2018)	-	-	-	-	-	888 (100%)
	IAC-PCI (N= 600) (Xie et al., 2013)	-	-	-	-	-	600 (100%)

**Prospective Genotyping (Observational Trials)**	IGNITE (N= 3,342) (Beitelshees et al., 2022)[#]	2,448 (73.2%)	659 (19.7%)	38 (1.1%)	118 (3.5%)	73 (2.2%)	124 (3.7%)
	GIANT (N= 1,445) (Hulot et al., 2020)	-	-	-	-	-	1,445 (100%)
	PHARM-ACS (N=1,361) (Zhang et al., 2021)	-	-	1,334 (98.0%)	-	-	27 (2.0%)
	Sánchez-Ramos et al (N=719) (Sánchez-Ramos et al., 2016)	-	-	-	-	-	719 (100%)
	Shen et al (N=628) (Shen et al., 2016)	-	-	-	-	-	628 (100%)

Other Race* refers to the number of individuals in the clinical study with race or ethnicity reported as Other in the study publication, which could include patients that identify as Native Hawaiian or Pacific Islander, American Indian or Alaska Native, or more than one race. Note that definitions for this classification may vary between studies.

^ In TAILOR-PCI, 1,187 (22.5%) participants were of East Asian ancestry and 236 (4.5%) were of South Asian ancestry

[#]The IGNITE numbers for all race and ethnicity groups were not included in the cited publication but were collected and provided by the IGNITE authors.
